# Dynamic changes in cellular atlases and communication patterns within yak ovaries across diverse reproductive states unveiled by single-cell analysis

**DOI:** 10.3389/fcell.2024.1444706

**Published:** 2024-08-29

**Authors:** Jie Pei, Lin Xiong, Xingdong Wang, Shaoke Guo, Mengli Cao, Ziqiang Ding, Yandong Kang, Min Chu, Xiaoyun Wu, Pengjia Bao, Xian Guo

**Affiliations:** ^1^ Key Laboratory of Yak Breeding in Gansu Province, Lanzhou Institute of Husbandry and Pharmaceutical Sciences, Chinese Academy of Agricultural Sciences, Lanzhou, Gansu, China; ^2^ Key Laboratory of Animal Genetics and Breeding on Tibetan Plateau, Ministry of Agriculture and Rural Affairs, Lanzhou, Gansu, China

**Keywords:** yak, ovary, reproductive state, single-cell, cellular atlas, cell communication

## Abstract

Yaks (*Bos grunniens*) exhibit exceptional adaptation to the challenging high-altitude environment of the Qinghai-Tibetan plateau, making them the sole bovine species capable of thriving in such exreme conditions. Investigating the cellular and molecular characteristics of yak ovaries across different reproductive states is crucial for gaining insight into their ovarian functions. Herein, the cellular atlases of yak ovaries in different reproductive states were depicted by single-cell RNA-sequencing (scRNA-seq). The cellular atlases of the ovaries were established by identifying specific gene expression patterns of various cell types, including granulosa cells, theca cells, stromal cells, smooth muscle cells, endothelial cells, glial cell, macrophages, natural killer cells, and proliferating cells. The cellular compositions of the ovaries vary among different reproductive states. Furthermore, the granulosa cells comprise six cell subtypes, while theca cells consist of eight cell subtypes. The granulosa cells and theca cells exhibit distinct biological functions throughout different reproductive states. The two cell types were aligned along their respective pseudotime trajectories. Moreover, a cell-to-cell communication network was constructed among distinct cell types within the ovary, spanning the three reproductive states. Notably, during the estrus period, the granulosa cells demonstrated more prominent interactions with other cell types compared to the remaining reproductive states.

## 1 Introduction

Yak (*Bos grunniens*) is remarkable creature that has evolved exceptional adaptations enabling them to thrive in the extremely challenging environment of the Qinghai-Tibetan plateau in China (Mizuno et al., 2015; Wu, 2016). The plateau, known as the “Roof of the World,” is renowned for its high altitude, extreme cold and low oxygen levels, which pose significant challenges to life in this region (Qiu, 2007; Graham, 2023). The yak plays a vital role in supporting the production and livelihood of the nomadic communities living in the plateau regions, serving as a source of food, shelter, fuel, and transportation (Lensch, 1996; Guo et al., 2014). Reproductive success plays a crucial role in the survival and population dynamics in yak. However, female yaks’ reproductive efficiency is greatly influenced by seasonal mating behavior, which is heavily dependent on environmental factors (Zi, 2003). Female yaks reach sexual maturity and breed seasonally, exhibiting oestrus from July to September (Prakash et al., 2008). The gestation period lasts from September to June of the following year, while some may experience anoestrus during this time (Yu et al., 1993).

Ovary is vital for female reproduction (Edson et al., 2009), executing its biological functions as both an endocrine organ capable of producing hormones such as sex hormones (DeFalco and Capel, 2009; Phelan and Conway, 2011) and a reproductive organ producing and releasing the mature oocyte (De Vos et al., 2010; Gheorghisan-Galateanu et al., 2014; Frost et al., 2023; Mantri et al., 2023). The ovary undergoes significant changes throughout a female reproductive life, including during puberty, the menstrual cycle, pregnancy, and menopause in human (Mihm et al., 2011). For domestic animals, reproductive cycle is largely categorised into oestrous and pregnant or anoestrous periods, which provides a convenient framework for monitoring animal production. Moreover, the oestrous period can be divided into prooestrous, oestrous, metoestrous, and dioestrous phases, each phase is characterized by unique endocrine profiles (Jaimes et al., 2019; Suman and Lino de Oliveira, 2022; Piibor et al., 2023). Due to the complex nature of ovarian function, which changes dynamically during reproductive cycle, it has historically been challenging to elucidate the cell-type-specific mechanisms that promote alteration of normal ovarian responsibility. The reproductive performance of the ovary relies on its cellular composition and molecular characteristics, which collectively determine its normal ovarian functions. Therefore, is essential to delineate the cellular and molecular dynamics of the ovary during the reproductive cycle and the paracrine factors that help coordinate this process to gain insight into their ovarian functions.

Single-cell RNA-sequencing (scRNA-seq) has emerged as a powerful tool for characterizing cell types and gene expression patterns in complex tissues. Until now, scRNA-seq has uncovered the cellular molecular characteristics within ovary for some species. Cell atlases that provide a comprehensive understanding of cellular composition have been created for the species, including human (Fan et al., 2019; Wagner et al., 2020; Yan et al., 2023), monkey (Wang et al., 2020b; Zhao et al., 2020a), mouse (Zhao et al., 2020b; Meinsohn et al., 2021), *drosophila* (Fu et al., 2020; Jevitt et al., 2020; Rust et al., 2020), zebrafish (Liu et al., 2022), seabass (Liu et al., 2021), and teleost (Wang et al., 2021). Using surgical specimens, the mapping of cell types within the human ovary was conducted, and the cataloguing of transcriptomic changes during follicular development and regression was performed (Fan et al., 2019). The investigation of changes in ovarian cell types and states that occur with ageing has been conducted using a primate model (Wang et al., 2020b). Further, the process of follicle formation during early embryonic ovarian development was investigated to determine the relationship between oocytes and their supporting cells in the formation of follicles (Zhao et al., 2020b). The identification of inhibitory pathways regulated by anti-Mullerian hormone (AMH) was accomplished during the initial wave of follicular growth in the murine ovary (Meinsohn et al., 2021). While these studies do not account for the dynamic nature of cell states that occur throughout the reproductive cycle, including the oestrous cycle. In a recently published study, the cell types and their transcriptional dynamics within the mouse ovary during the oestrous cycle were elucidated (Morris et al., 2022). However, there is still a desperate need to decipher the dynamics of cell types and communication across various reproductive states on a larger time scale.

**FIGURE 1:**
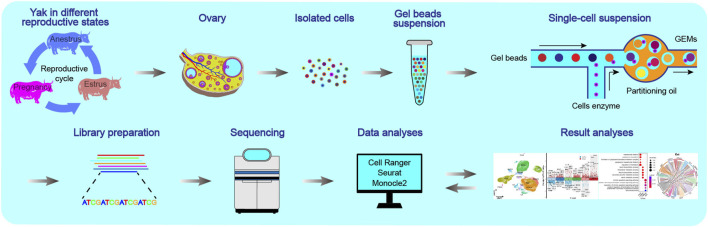
Schematics of the workflow of the single-cell transcriptome for yak ovaries. The ovaries were selected from yaks in three reproductive states: anoestrus, oestrus, and pregnancy.

To address the research gap, we performed scRNA-seq to investigate the cellular atlases of yak ovaries and their communication across different states of the reproductive cycle (Figure 1). Ovaries were harvested from yaks in the three states of the normal reproductive cycle including anoestrus, oestrus, and pregnancy. The utilisation of samples during pregnancy is of great significance in the investigation of ovarian function. Herein, we 1) decoded the cellular molecular dynamics within the ovaries during reproductive cycle, and identified signature genes that could potentially serve as biomarkers for specific cell types; 2) analysed the heterogeneity and developmental trajectories of granulosa cells and theca cells; 3) revealed variations in the cellular composition and biological functions of granulosa cells and theca cells throughout the different reproductive states; 4) constructed cell-to-cell communication networks among different cell types within the ovaries spanning the reproductive cycle. Overall, our study contributes to determining how ovary modulates its cellular phenotypes to adapt to different reproductive functions, understanding the mechanisms underlying successful reproduction, and developing reproductive approaches for improving reproductive health and lifespan in females.

## 2 Materials and methods

### 2.1 Animals

Due to the seasonal reproductive nature of yaks, ovaries in anoestrus, oestrus, and pregnancy were respectively collected in April, August, and December. The experimental subjects consisted of healthy female yaks, aged four to 5 years, free from reproductive diseases, living in Qinghai Province, China, at altitudes exceeding 3,200 m above sea level.

### 2.2 Ovary samples collecting during different reproductive states

The ovaries, free from anatomical abnormalities, were promptly removed from yak carcasses after slaughter. Subsequently, the fresh ovaries were rinsed with physiological saline to eliminate blood. Six ovaries from distinct individuals in the anoestrous group, seven from the oestrous group, and eleven from the pregnant group were selected for the HE staining experiment. Furthermore, three ovaries from each group underwent immunofluorescence staining, and two out of the three ovaries were chosen for scRNA-seq analysis.

### 2.3 Haematoxylin and eosin staining

After the yak ovaries were removed, they were rinsed with cold normal saline and then cut into approximately 5 mm cubed pieces using a sterile scalpel blade. The cubed pieces of ovarian tissue were promptly rinsed with phosphate-buffered saline (PBS) and fixed in 4% methanol-free paraformaldehyde. Following this, the ovarian tissue pieces underwent dehydration through successive incubations in ethanol and xylene. The dehydrated tissue pieces were then solidified using paraffin. Subsequently, the paraffin-embedded tissue pieces were sectioned into 4 μm sections and mounted onto slides. These sections were deparaffinised using successive xylene baths and rehydrated through a descending series of alcohol. Following the haematoxylin staining, the deparaffinised sections were treated with 0.3% acid alcohol and transferred into a 0.6% ammonium hydroxide aqueous solution until a blue colour appeared. Subsequently, the sections were immersed in 85% alcohol and stained with eosin. Afterward, the sections underwent the same dehydration series as described above. Finally, the sections of ovarian tissue were cleared in xylene, sealed with a neutral gum seal, and imaged using a microscope approximately 24 h after staining.

### 2.4 Single-cell dissociation pretreating

The fresh yak ovaries were cut into small cubes of approximately 3 mm in size using a sterile scalpel. Five cubes were obtained from each ovary and washed with PBS. Each cube was then transferred individually into a cryovial containing 1 mL of cell cryopreservation medium (80% DMEM, 10% DMSO, and 10% FBS), mixed well, and allowed to stand at room temperature (RT) for 10 min. Subsequently, the cryovials were placed in a freezing box containing sufficient isopropanol. The freezing box was immediately immersed in dry ice and subjected to gradual freezing for 12 h. Finally, the cryovials were stored in liquid nitrogen for long-term cryopreservation.

### 2.5 Single-cell dissociating

The ovarian tissue fragments underwent dissociation for scRNA-seq. Briefly, the cryopreserved tissue fragments were thawed in a water bath at 37°C and further minced into approximately 0.5 mm^3^ pieces in cooled RPMI 1640 medium containing 0.04% bovine serum albumin (BSA). The fragments were then digested using enzymes, including 1 mg/mL collagenase Type II (Life Technologies, Grand Island, NY, United States) and 0.25% trypsin-EDTA (Life Technologies, United States), overnight on ice. The digestion process was halted by adding DMEM supplemented with 10% foetal calf serum (Gibco, Paisley, United Kingdom). To collect the dissociated cells, the cell suspension was centrifuged at 160× g for 3 min. Subsequently, the dissociated cells were incubated with advanced DMEM/F12 Glutamax (Life Technologies) containing 1% insulin-transferrin-selenium mixture (Life Technologies, United States), 1% penicillin-streptomycin (Life Technologies, United States), and 27 IU/mL RNase-free DNase I (Qiagen, Hilden, Germany) at 37°C for 1 h. The cells were then resuspended in DPBS containing 2% FBS and passed through a 40 μm cell strainer (Corning, NY, United States) to eliminate any remaining cell aggregates.

### 2.6 Single-cell RNA library construction and sequencing

The cell suspension was processed to remove dead cells using a dead cell removal kit (MACS, Milteny Biotec, Bergisch Gladbach, Germany), resulting in single-cell suspensions containing predominantly viable cells. These suspensions were then loaded into chips of a Chromium Next GEM Single Cell 3′ Reagent Kit v3.1 (PN-1000128, 10× Genomics, Pleasanton, CA, United States) and subjected to a Chromium Single Cell Controller (10× Genomics, United States) to create single-cell GEMs. A primer containing Illumina TruSeq Read 1 sequence, a 10× Barcode, a UMI, and a poly-dT sequence was utilised to generate barcoded full-length cDNA from polyadenylated messenger RNA. After the GEMs were broken and the pooled fractions recovered, the barcoded first-strand cDNAs were purified with silane magnetic beads and then amplified to produce sufficient mass for library construction. Enzymatic fragmentation and size selection were employed to optimize cDNA amplicon size. The addition of P5, P7, a sample index, and a TruSeq Read 2 to the amplicon was accomplished through end repair, A-tailing, adaptor ligation, and PCR. Subsequently, library synthesis and RNA-seq were performed using an Illumina sequencing platform (Novaseq 6,000) with a 300 cycles kit (Illumina, San Diego, CA, United States), producing paired-end reads of 150 bp.

### 2.7 Single-cell data matrix quality controlling

The raw Chromium scRNA-seq data underwent processing through the Cell Ranger pipeline, supplied by 10× Genomics (v2.2.0), with reads being aligned to the BosGru3.0 yak genome utilising the STAR aligner (Dobin et al., 2013). This process resulted in the creation of cellular gene expression matrices containing valid cell barcodes and genes. The cellular gene expression matrices were converted into Seurat objects in the R environment (version 4.2.3, Vienna, Austria) using the Seurat package version 4.4.0 (Stuart et al., 2019). The cells expressing over 200 genes and genes expressed in a minimum of cells were retained for subsequent bioinformatics analyses.

In order to improve the quality of expression matrices, the “PercentageFeatureset” function was utilised to calculate the proportions of hemoglobin, mitochondrial, and ribosomal features. To filter out low-quality cells and exclude cells with extreme values indicating low complexity, duplets, or apoptotic cells, cells with hemoglobin genes, and those with unique feature counts exceeding 2,500 or falling below 200, as well as those with a percentage of mitochondrial genes out of the total gene count higher than 20%, were excluded from the analysis.

The remaining data were normalized using the “NormalizeData” function from the Seurat package in R. Next, we identified the top 2,000 genes with high variation using the Seurat function “FindVariableFeatures” with the method of variance stabilisation transformation (Macosko et al., 2015). The matrices were incorporated into the current Seurat object to create a new integrated dataset using the “FindIntegrationAnchors” and “IntegrateData” functions. The gene variables were centred using the “ScaleData” function.

### 2.8 Dimensionality reducing, cell clustering, and marker gene finding

The scaled data underwent dimensionality reduction using Seurat’s “RunPCA” function. The number of PCs was determined using the “ScoreJackStraw” and “Elbowplot” functions to identify the optimal condition for clustering. Cell clustering was performed using the “FindNeighbors” and “FindClusters” functions. 18 PCs were selected as the dataset’s dimensionality. The Louvain algorithm was used to optimise modularity in cell clustering, with a resolution parameter of 0.8 selected after parameter optimisation ranging from 0.4 to 1.2. DEGs were identified using the “FindAllMarkers” function (Wilcoxon Rank Sum test). During this process, a base 2 logarithm fold change threshold of 0.25 and a minimum percentage of cells expressing specific genes set at 0.25 were applied.

### 2.9 Cell type annotating

GO and KEGG enrichment analyses were performed on the top 200 variable DEGs in each cell cluster using the Bioconductor packages “clusterProfiler” (Yu et al., 2012) and “org.Bt.eg.db.” Cell clusters were annotated based on highly variable genes within each cluster using the “SingleR” package. Further annotation of the clusters into specific cell types was done using the CellMarker dataset as a reference, relevant previous studies (Man et al., 2020; Morris et al., 2022), GO enrichment results, and the “SingleR” annotation outcomes. To ensure the accuracy of cell annotation, GO and KEGG enrichment analyses were also performed on the top 200 variable DEGs in each cell type.

### 2.10 Validating through immunofluorescence

The yak ovarian tissue was prepared by cutting it into approximately 5 mm cubed pieces. These pieces were fixed overnight in 4% methanol-free paraformaldehyde. Following fixation, the tissue was transitioned to 70% ethanol and embedded in paraffin using a Shandon Excelsior tissue processor from Thermo Scientific. After embedding, the paraffin-embedded tissue blocks were sectioned into slices of 4 μm in thickness using an RM2065 microtome manufactured by Leica Instruments GmbH. These sections were mounted onto StarFrost slides and then subjected to immunostaining. For immunostaining, the paraffin sections were deparaffinised and rehydrated. Antigen retrieval was carried out in a steamer using 0.01 M sodium citrate buffer (pH 6.0) for 20 min. Subsequently, the slides were then blocked with a blocking buffer containing 1% BSA and 0.05% Tween-20 in PBS at RT for 1 h. The tissue sections were then incubated with primary antibodies including rabbit anti-RGS5 (1:500, CPA2283, Cohesion), anti-PECAM1 (1:200, bs-0195R, Bioss, Beijing, China), anti-AIF1 (1:100, NB100-1028SS, NOVUS), anti-CYP11A1 (1:200, bs-10099R, Bioss, Beijing, China), and anti-MIS18BP1 (1:200, bs-9615R, Bioss, Beijing, China) at 4°C overnight. The following day, the sections were incubated with a secondary antibody, specifically goat anti-rabbit IgG (1:500, A16118, ThermoFisher, Waltham, MA, United States) at RT for 1 h. After that, the sections were counterstained with 4′,6-diamidino-2-phenyl-indole (DAPI, Life Technologies, United States), and mounted with ProLong Gold (Life Technologies, United States) in fluorescent mounting media (Dako Agilent). The immunostained slides were then scanned using a Pannoramic 250 Flash III digital scanner (3DHISTECH Ltd., located in Budapest, Hungary), and representative areas were selected for imaging through the utilisation of “SlideViewer” software (version 2.5.0, 3DHISTECH Ltd.).

### 2.11 Granulosa cells and theca cells heterogeneity analysing

The gene expression matrices for granulosa cells and theca cells were individually extracted from the overall gene expression matrix. Similar processes were applied to analyse the heterogeneity of both cell types. The gene expression values were standardized by centring them to a mean of zero and scaling them to a standard deviation of one. Following this, PCA was utilised for dimensionality reduction to identify the optimal number of PCs for clustering. Subsequently, cell clustering was performed to distinguish subtypes of granulosa cells and theca cells, using 18 PCs and a resolution parameter set to 0.4. DEGs were identified for each cell subtype. Furthermore, GO and KEGG enrichment analyses were conducted on the top 200 DEGs of each cell subtye to explore the potential functions of the cell subtypes.

### 2.12 Developmental trajectory for granulosa cells and theca cells constructing

To explore the continuous progression of cell states in granulosa cells and theca cells, a pseudotime trajectory analysis was conducted using the R package “monocle” (Qiu et al., 2017). This analysis was based on the transcriptional dynamics observed in these cell types. Monocle objects were created from the Seurat objects of granulosa cells and theca cells separately, utilising the function “newCellDataSet” from the “monocle” package with a lowerDetectionLimit of 0.5. Similar methodologies were applied to analyse the developmental trajectories of both cell types. The DDRTree algorithm was utilised to visualise the pseudotime trajectory in a reduced dimensional space.

### 2.13 Cell communication analysis

Cell-cell communication via ligand-receptor interactions was explored using the Python package “CellPhoneDB” (Efremova et al., 2020) to investigate potential interactions between different cell types. The presence of known ligand-receptor pairs in the cell types was analysed to infer the cell-cell interplays. An interaction between two cell types was considered to exist if a receptor and its ligand were expressed in at least 10% of the corresponding tested cell population. Bubble plots were generated to visualise the significant ligand-to-receptor interactions between granulosa cells and other cell types, enabling the examination of cell communication differences between granulosa cells and other cell types across different reproductive states.

### 2.14 Quantification and statistical analysis

For GO analysis a significance threshold of *p* = 0.05 was used after adjusting the *p*-value using the Benjamini–Hochberg procedure implemented in the clusterProfiler R-software package. All antibody immunofluorescence experiments have been performed at least two times using identical or varying antibody combinations. Antibody immunofluorescent experiment were carried out on tissue samples of at least three individual yaks.

## 3 Results

### 3.1 Ovarain morphological and histological characteristics in different reproductive states

**FIGURE 2:**
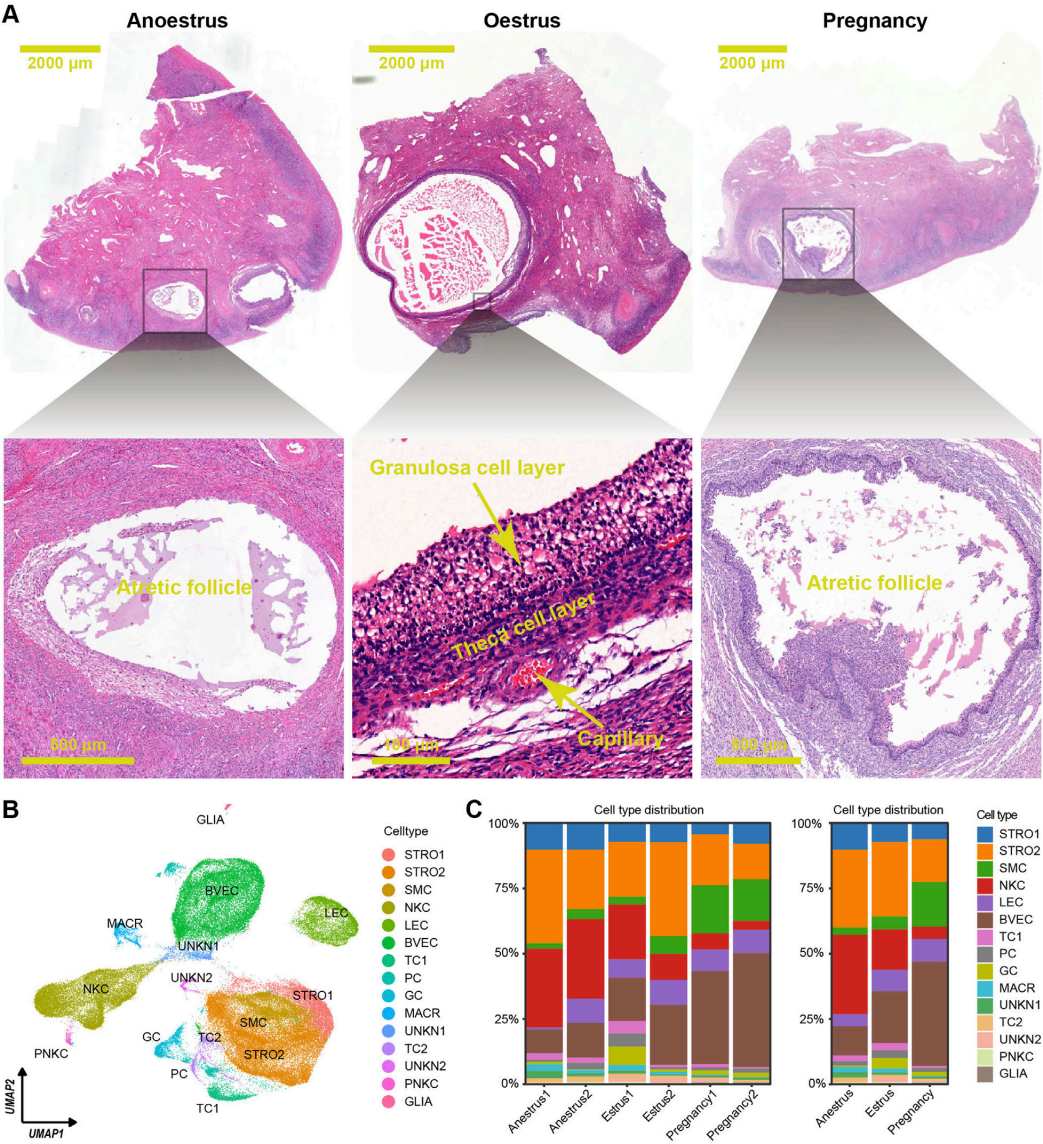
Histological features and cell type distributions within yak ovaries across different reproductive states. **(A)** Haematoxylin and eosin-stained sections exhibiting morphological and histological characteristics of yak ovaries from the different reproductive states. The scale bars in the top three pictures are set at 2,000 μm, while the scale bars in the bottom pictures are set at 500 μm, 100 μm, and 500 μm respectively. Ovaries from anoestrous and pregnant yaks usually possess many follicles under 2 mm in diameter, and most of them are in atretic state. In contrast, ovaries from oestrous yaks generally have a dominant follicle above 3 mm in diameter which is in developmental period. **(B)** Uniform manifold approximation and projection (UMAP) scatterplot visualising various cell types within all yak ovaries. Each point corresponds to a single-cell is colour-coded according to its cell type membership. STRO, stromal cell; SMC, smooth muscle cell; NKC, natural killer cell; LEC, lymphatic endothelial cell; BVEC, blood vascular endothelial cell; TC, theca cell; PC, proliferating cell, GC, granulosa cell; MACR, macrophage; PNKC, proliferating natural killer cell; GLIA, glial cell; UNKN, unknown cell. **(C)** Stacked bar charts demonstrating cell type distribution within yak ovaries among individuals (left) and different reproductive states (right).

To analyse the ovarian variations among yaks in different reproductive states, six ovaries were collected during anoestrus, seven ovaries during oestrus, and eleven ovaries during pregnancy periods. Morphological observations revealed that during oestrus in yaks, a dominant follicle with a diameter of over 5 mm was commonly observed extruding from the ovarian surface, along with several smaller follicles. In contrast, during anoestrus and pregnancy, many follicles with diameters under 5 mm were observable on the ovarian surface. Histological sections colouration with haematoxylin and eosin (HE) indicated that during oestrus in yaks, an increased presence of developing follicles within the ovarian tissue was observed. Conversely, in both anoestrus and pregnancy, we observed similarities in the follicle quantity, with a decrease in developing follicles and an increase in atretic follicles exhibiting a disorderly arrangement of the granulosa cell layer (Figure 2A), which aligns with our previous research findings (Guo et al., 2021).

### 3.2 Integration of single-cell RNA sequencing data

To study cell type diversity, scRNA-seq dataset were generated from six ovaries, with two ovaries collected from yaks in each of the anoestrous, oestrous, and gestational periods. The individual scRNA-seq datasets were integrated, resulting in the creation of a comprehensive Seurat file consisting of 79,776 cells expressing 19,488 genes.

### 3.3 Cell clusters, cell types, and marker genes

The scRNA-seq dataset was effectively normalized and centred. The process of dimensionality reduction for cellular gene expression was executed seamlessly, identifying the top 18 principal components (PCs) as the optimal PCs for subsequent clustering analyses. The ovarian cells were separated into 25 unique clusters (Supplementary Figure S1A). The differentially expressed genes (DEGs) were selected from each cluster using adjusted *p*-values determined by the Wilcoxon rank sum test (Supplementary Table S1; Supplementary Figure S1B). Based on the unique DEG profiles and their corresponding biological functions within each cluster (Supplementary Table S2; Supplementary Figure S2), manual annotation was conducted. Then, successful annotation resulted in the classification of the 25 clusters into 15 distinct cell types including granulosa cells, theca cells, theca cells, stromal cells, stromal cells, blood vascular endothelial cells, lymphatic endothelial cells, smooth muscle cells, natural killer cells, proliferating natural killer cells, macrophages, glial cells, proliferating cells, unknown cells, and unknown cells (Supplementary Table S3; Supplementary Figure S3). Each cell type underwent DEG filtering (Supplementary Table S4), followed by enrichment analysis of biological functions based on the resulting DEGs (Supplementary Table S5; Supplementary Figure S4), confirming the accuracy of cell type annotation. A two-dimensional plot was created using uniform manifold approximation and projection (UMAP) to visualise the cell types (Figure 2B).

**FIGURE 3:**
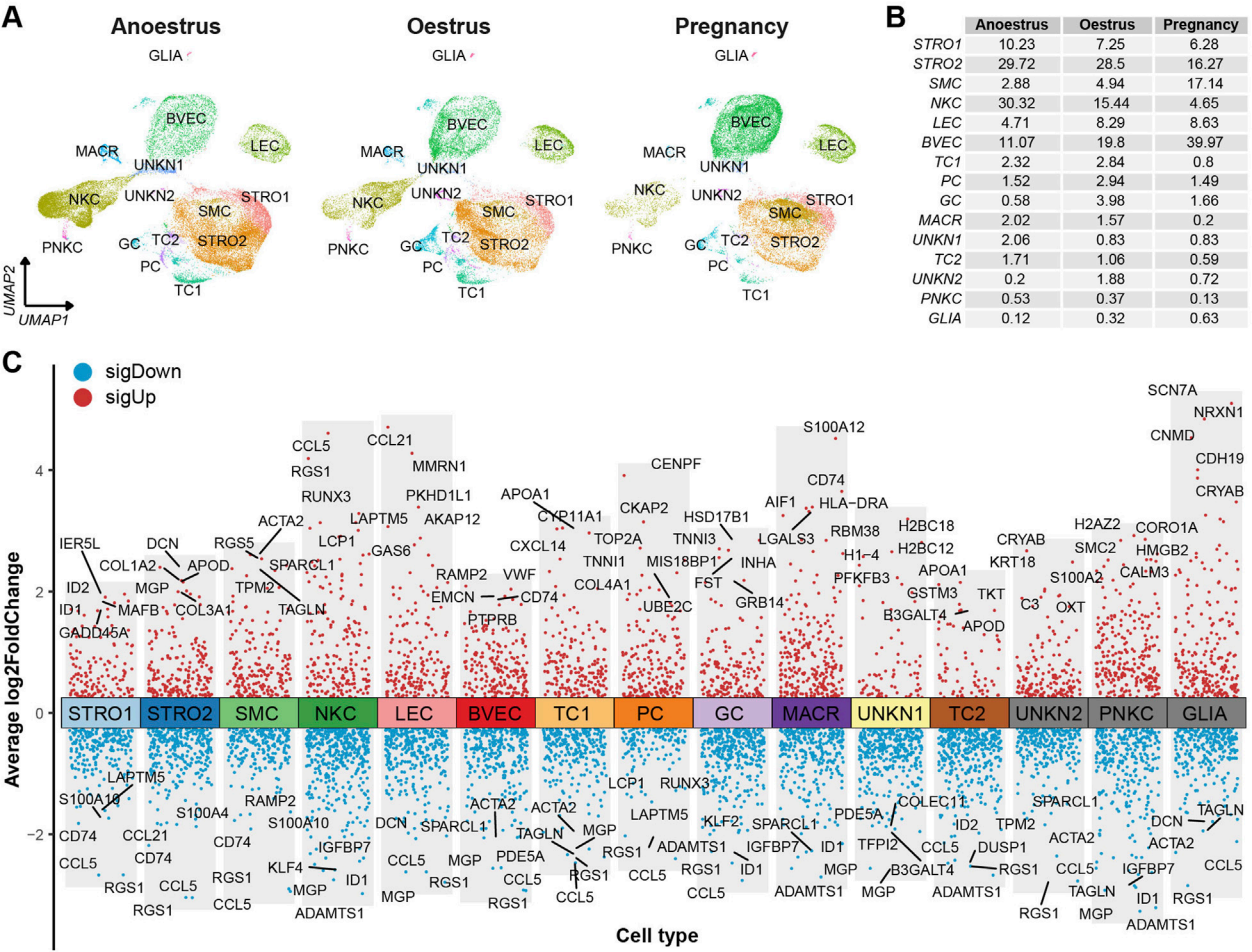
Cell type distributions and their signature genes within yak ovaries. **(A)** UMAPs showing cell type distributions for yak ovaries of different reproductive states. STRO, stromal cell; SMC, smooth muscle cell; NKC, natural killer cell; LEC, lymphatic endothelial cell; BVEC, blood vascular endothelial cell; TC, theca cell; PC, proliferating cell; GC, granulosa cell; MACR, macrophage; PNKC, proliferating natural killer cell; GLIA, glial cell; UNKN, unknown cell. **(B)** A table exhibiting cell percentages within yak ovaries of different reproductive states. **(C)** Volcano plot visualising differentially expressed genes (DEGs) among cell types. Top 5 highly and lowly expressed DEGs are labeled.

The consistent distribution of cell types within each group, as reflected by similar percentages, indicates the reliability and consistency of the sampling procedure (Figure 2C left). However, the distributions of cell types vary across the different reproductive states (Figure 2C right). There is a significant difference in the number of immune cells, such as natural killer cells and macrophages, between the ovaries of yaks during anoestrus and the other groups. The former contains a higher number of these immune cells (Figures 3A, B; Supplementary Table S6). In comparison to the other groups, the ovaries of oestrous yaks exhibit a higher abundance of granulosa cells and proliferating cells. The presence of smooth muscle cells and blood vascular cells in the ovaries of pregnant yaks is significantly higher compared to the other groups.

**FIGURE 4:**
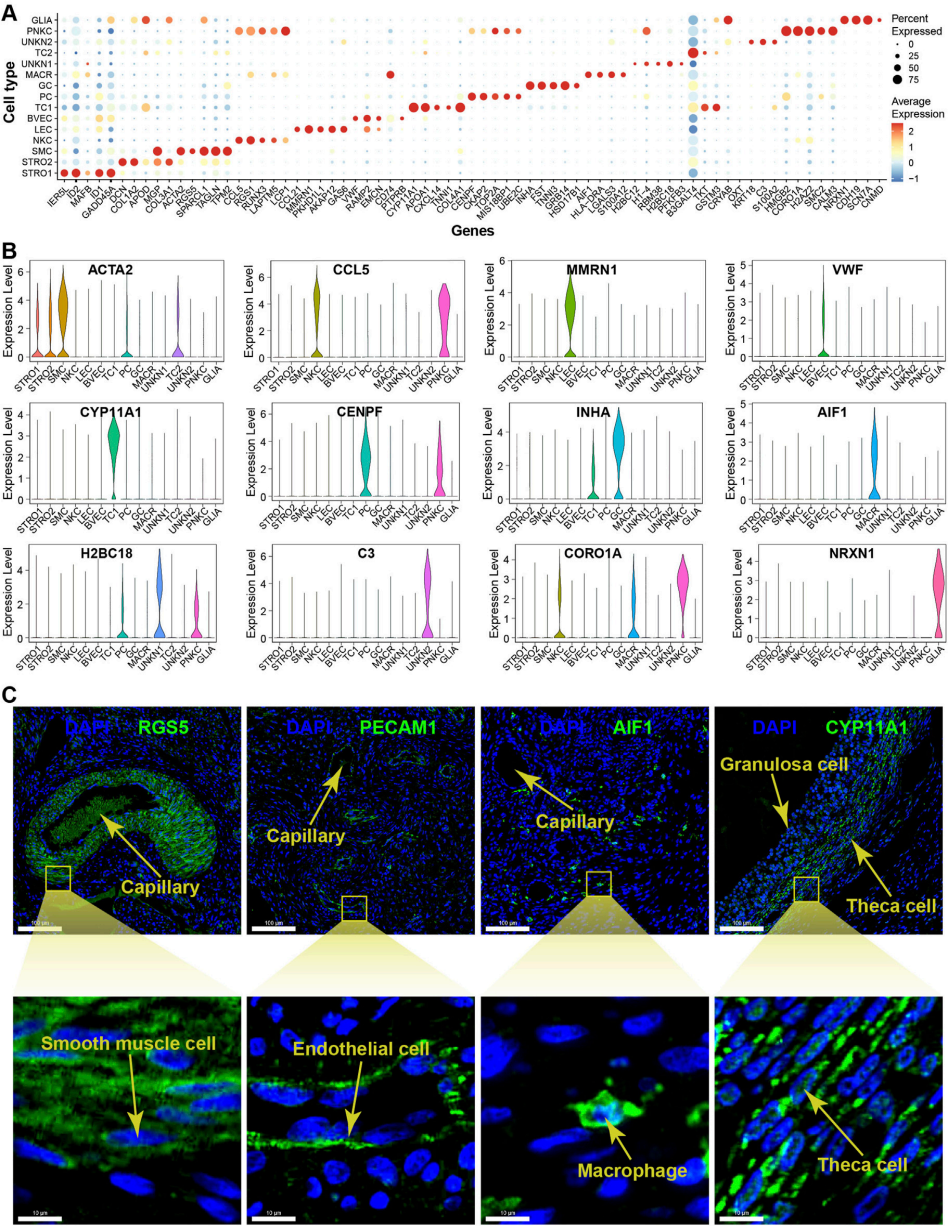
Expression specificity of signature genes among cell types within yak ovaries. **(A)** Dot plot showing expression patterns of top 5 highly expressed genes within each cell type. Dot size represents the percentage of cells expressing a specific gene. Colour gradient from blue to red indicates gene expression levels from low to high. STRO, stromal cell; SMC, smooth muscle cell; NKC, natural killer cell; LEC, lymphatic endothelial cell; BVEC, blood vascular endothelial cell; TC, theca cell; PC, proliferating cell; GC, granulosa cell; MACR, macrophage; PNKC, proliferating natural killer cell; GLIA, glial cell; UNKN, unknown cell. **(B)** Violin plots visualising the expression specificity of signature genes across cell types. The vertical axis represents the expression scores of the signature genes. The expression values of the signature genes were adjusted using log-normalization. **(C)** Sections detected by immunofluorescence validating the expression of RGS5, PECAM1, AIF1, and CYP11A1 as markers for smooth muscle cells, endothelial cells, macrophages, and theca cells, respectively. DAPI, a fluorescent stain, was employed as a nuclear counterstain. The scale bars in the top pictures are set at 100 μm, while the scale bars in the bottom pictures are set at 10 μm.

The DEGs filtered from each cell type based on their adjusted *p*-values are demonstrated in a volcano plot, highlighting the top and bottom five DEGs with the highest and lowest expression levels across all cell types (Figure 3C). Meanwhile, a dot matrix visualisation illustrates the expression levels and percentages of the top five genes across different cell types, emphasizing the presence of specific DEGs within each cell type (Figure 4A). The majority of DEGs exhibit specific expression patterns within their respective cell types.

The violin plots illustrate the expression specificities of the most representative DEGs that can be considered as potential marker genes specific to each cell type, such as ACTA2, CCL5, MMRN1, VWF, CYP11A1, CENPF, INHA, AIF1, H2BC18, C3, CORO1A, and NRXN1 (Figure 4B). The expression specificities of the signature genes RGS5, PECAM1, AIF1, and CYP11A1 were confirmed using immunofluorescence technique for smooth muscle cells, endothelial cells, macrophages, and theca cells, respectively. The magnified immunofluorescence section clearly demonstrates that the specific expression of each signature gene can be specifically detected in their respective cell types (Figure 4C). In more detail, the marker genes RGS5 and PECAM1 are primarily expressed in the cytoplasm of smooth muscle cells and endothelial cells, respectively. In contrast, the marker genes AIF1 and CYP11A1 exhibit whole-cell expression patterns in macrophages and theca cells, respectively.

### 3.4 Heterogeneity of granulosa cells

**FIGURE 5:**
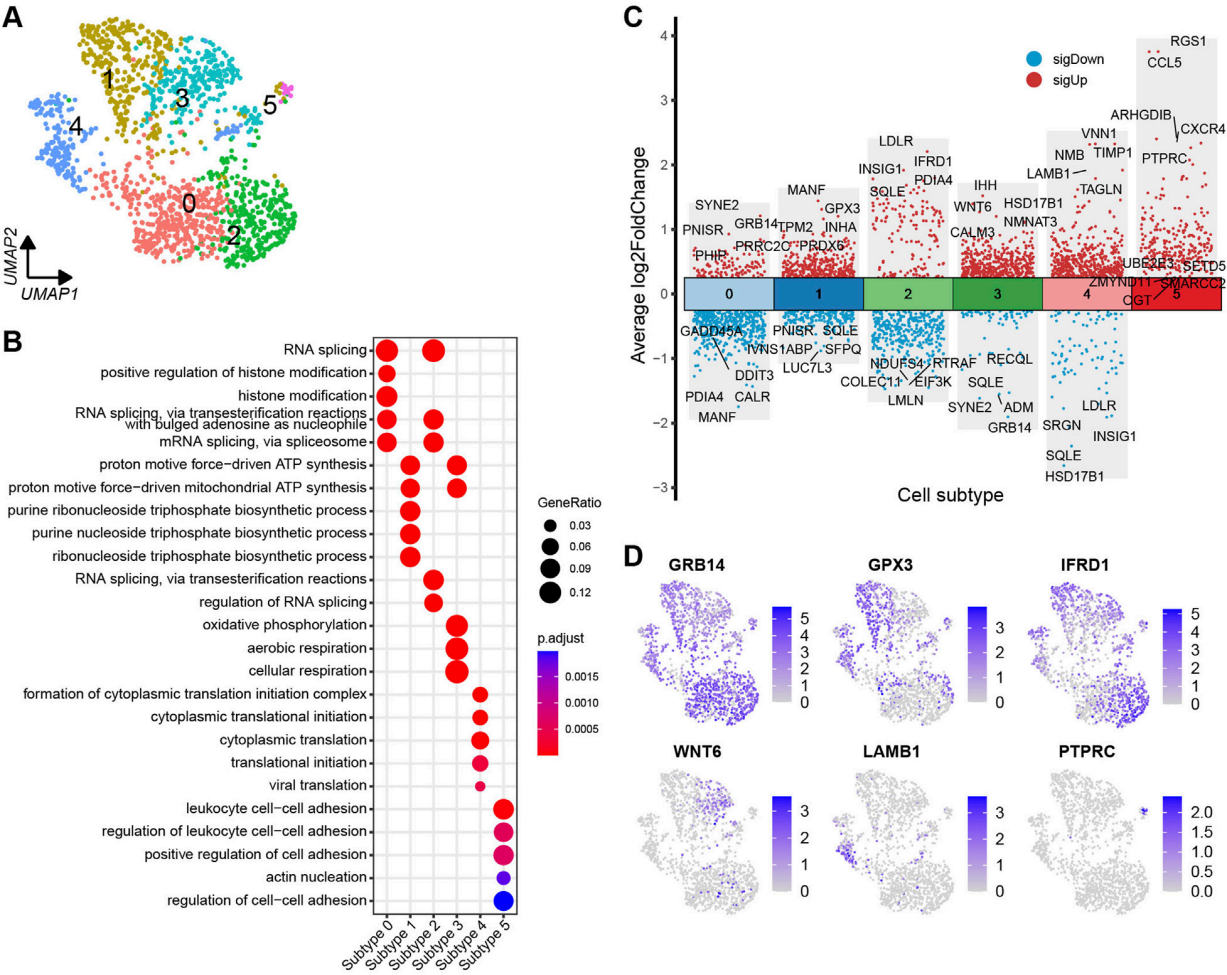
Heterogeneity of granulosa cells in yak ovaries. **(A)** Uniform manifold approximation and projection (UMAP) scatterplot visualising diverse cell subtypes of granulosa cells. **(B)** Bubble plot exhibiting top 5 enriched biological processes terms from gene ontology analysis for each granulosa cell subtype. Bubble size represents gene ratio in the corresponding biological process. Colour gradient from red to blue represents adjusted p-values from low to high. **(C)** Volcano plot visualising DEGs among granulosa cell subtypes. Top 5 highly and lowly expressed DEGs are labeled. **(D)** Feature plots demonstrating the expression specificity of signature genes among granulosa cell subtypes. Colour gradient from light gray to blue indicates gene expression levels from low to high.

The granulosa cells were further categorised into six distinct cell subtypes based on the DEGs identified across the subtypes (Figure 5A). A volcano plot visualises the top and bottom five DEGs with the highest and lowest expression levels across granulosa cell subtypes (Figure 5C). The majority of DEGs exhibit specific expression patterns within their respective cell types. Further, UMAP plots highlight specific genes associated with each cell subtype, such as GRB14, GPX3, IFRD1, WNT6, LAMB1, and PTPRC, which can be considered as marker genes for their respective cell subtypes (Figure 5D). To identify the functions of each granulosa cell subtype, GO (Gene ontology) enrichment analysis was performed using the DEGs specific to each subtype. A more comprehensive understanding of the distinctive functional characteristics of each subtype has been acquired. The enriched functional categories for each granulosa cell subtype are displayed in a bubble plot (Figure 5B; Supplementary Table S7).

### 3.5 Heterogeneity of theca cells

**FIGURE 6:**
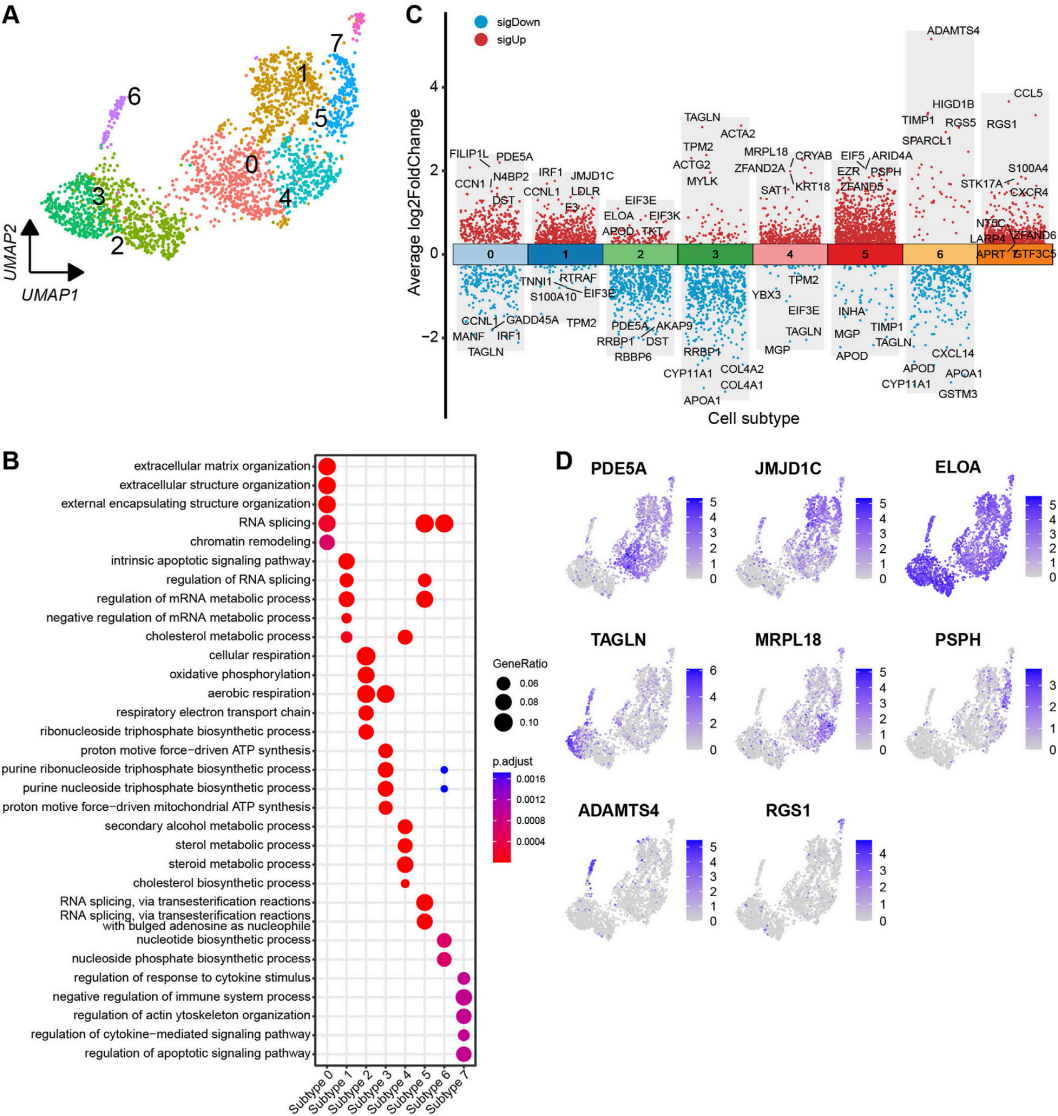
Heterogeneity of theca cells in yak ovaries. **(A)** Uniform manifold approximation and projection (UMAP) scatterplot visualising diverse cell subtypes of theca cells. **(B)** Bubble plot exhibiting top 5 enriched biological processes terms from gene ontology analysis for each theca cell subtype. Bubble size represents gene ratio in the corresponding biological process. Colour gradient from red to blue represents adjusted p-values from low to high. **(C)** Volcano plot visualising DEGs among theca cell subtypes. Top 5 highly and lowly expressed DEGs are labeled. **(D)** Feature plots demonstrating the expression specificity of signature genes among theca cell subtypes. Colour gradient from light gray to blue indicates gene expression levels from low to high.

Based on the DEGs identified across the subtypes, the theca cells are classified into eight distinct subtypes (Figure 6A). To visualise the DEGs, a volcano plot highlights the top five and bottom five DEGs with the highest and lowest expression levels across the theca cell subtypes (Figure 6C). Most of the DEGs show specific expression patterns within their respective cell types. Additionally, UMAP plots are employed to identify specific genes associated with each cell subtype. Noteworthy genes include PDE5A, JMJD1C, ELOA, TAGLN, MRPL18, PSPH, ADAMTS4, and RGS1, which could serve as marker genes for their respective cell subtypes (Figure 6D). In order to determine the functions of each theca cell subtype, DEGs specific to each subtype were subjected to a GO enrichment analysis. The resulting enriched GO terms for each theca cell subtype provided a more comprehensive understanding of their unique functional characteristics. A bubble plot is used to visualise these enriched functional categories (Figure 6B; Supplementary Table S8).

### 3.6 Pseudotime analysis on granulosa cells and theca cells

Developmental trajectories of the granulosa cells and theca cells were established to explore the transcriptomic pathways involved in their differentiation processes. Heat maps depicting the expression patterns of representative genes in the granulosa cells and theca cells reveal their temporal and progressive dynamics across pseudotime (Supplementary Figures S5A, S6A). The expression patterns of the signature genes among the cell subtypes of the granulosa cells and theca cells along the pseudotime axises show similar trends to those observed in the heat maps (Supplementary Figures S5B, S6B).

The pseudotimes of granulosa cells and theca cells are determined by their positions mapped along the principal curves, offering valuable insight into their developmental trajectory (Supplementary Figures S5C, D, S6C, D). The developmental trajectory of the granulosa cells begins in subtype 0 of theca cells, progresses through subtypes 1 and 3 in sequence, and culminates in subtype 2, with additional branches extending to subtypes 4 and 5. The granulosa cells from the ovaries of oestrous yaks encompass the entire developmental trajectory, while those from anoestrous yaks are only found at the branches of the trajectory. Granulosa cells from pregnant yaks are situated at the trunk and some parts of the branches (Supplementary Figure S5E). The developmental trajectory of the theca cells commences in subtype 2 of theca cells, advances sequentially through subtypes 3, 4, 6, and 7, and concludes in subtypes 1 and 5, with additional branches extending to subtype 0. The entire developmental trajectory is represented by the theca cells from the ovaries of anoestrous and oestrous yaks, while granulosa cells from pregnant yaks are located at the central axis and certain parts of the branches (Supplementary Figure S6E).

### 3.7 Granulosa and theca cell heterogeneity in different reproductive states

**FIGURE 7:**
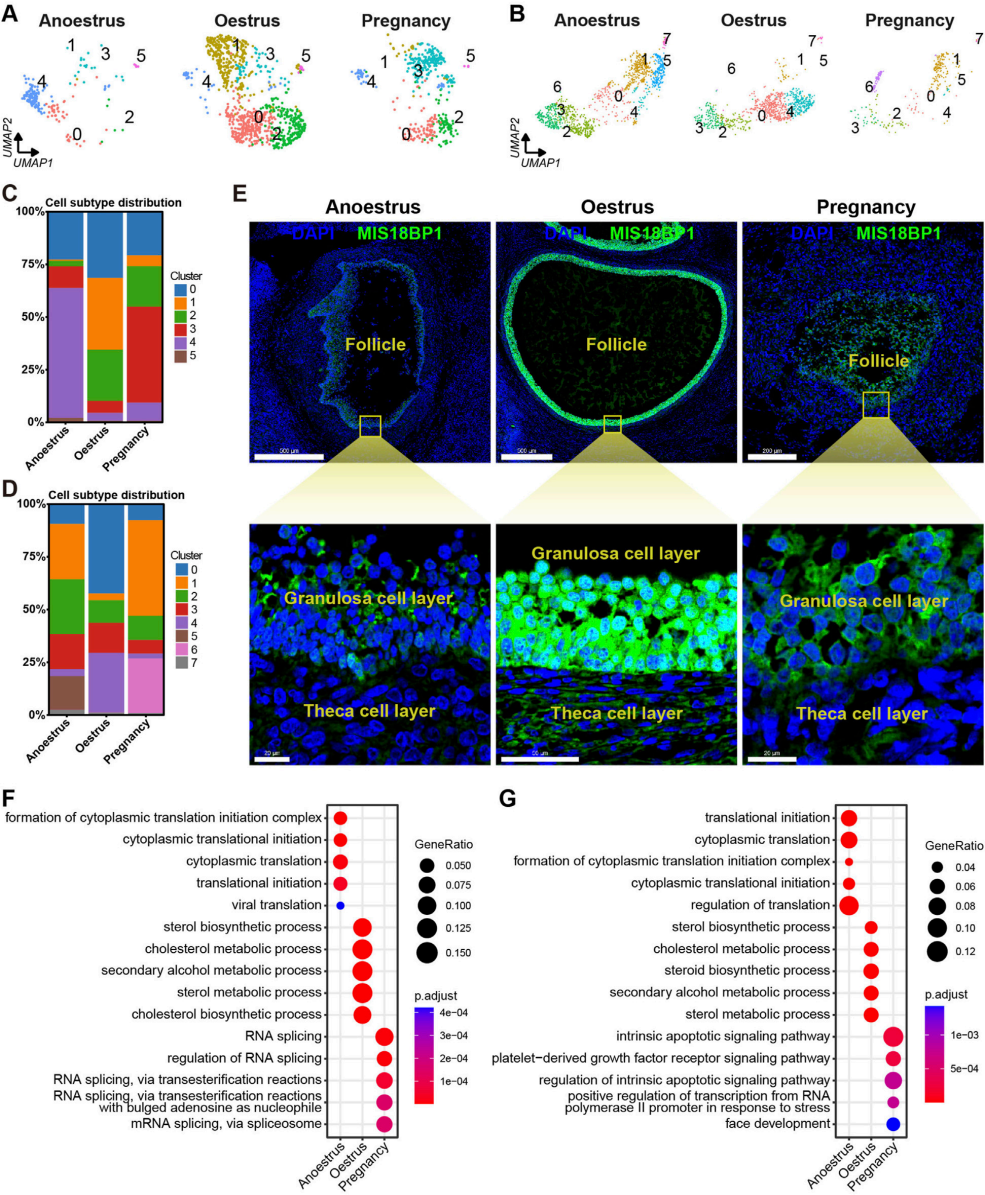
Heterogeneity of granulosa cells and theca cells across different reproductive states. **(A,B)** UMAPs showing cell subtype distributions of granulosa cells **(A)** and theca cells **(B)** across different reproductive states. **(C,D)** Stacked bar charts demonstrating cell subtype distributions of granulosa cells **(C)** and theca cells **(D)** across different reproductive states. **(E)** MIS18BP1 level on ovarian tissue sections detected by immunofluorescence revealing proliferative statuses of granulosa cells and theca cells in different reproductive states. DAPI, a fluorescent stain, was employed as a nuclear counterstain. The scale bars in the top three pictures are set at 500 μm, 500 μm, and 200 μm, while the scale bars in the bottom three pictures are set at 20 μm, 50 μm, and 20 μm. **(F,G)** Bubble plots exhibiting top 5 enriched biological processes terms from gene ontology analysis for each granulosa cell subtype **(F)** and theca cell subtype **(G)**.

The number of granulosa cells in the ovaries of anoestrous yaks is significantly higher compared to those in other reproductive states (Figures 3B, 7A). In anoestrous yaks, the ovaries contain a higher number of subtype 4 granulosa cells. In contrast, the ovaries of oestrous yaks have a greater abundance of granulosa cells belonging to subtypes 0, 1, and 2. Additionally, the ovaries of pregnant yaks exhibit a higher concentration of granulosa cells of subtype 3 (Figure 7C). The quantity of theca cells in the ovaries of pregnant yaks is notably lower than those in the other reproductive states (Figures 3B, 7B). During anoestrus, the ovaries of yaks have a higher presence of subtype 1 and 2 theca cells, while in oestrus, subtype 0 and 4 theca cells are more abundant. Moreover, pregnant yaks display a higher concentration of subtype 1 and 6 theca cells in their ovaries (Figure 7D).

To investigate the proliferative state, the expression levels of MIS18BP1 protein within the granulosa cells and theca cells were identified through immunofluorescence experiments. The expression level of MIS18BP1 is notably higher within the granulosa cells and theca cells during oestrus compared to those during the other reproductive states, with a particularly pronounced increase observed in the granulosa cells (Figure 7E). This result is consistent with the fact that follicles consisting of granulosa cells and theca cells are in a proliferative state during oestrus.

To investigate the functional heterogeneity of granulosa and theca cells in anoestrous, oestrous, and pregnant states, GO enrichment analysis of DEGs was conducted within these two cell types. The bubble plots displayed the top five biological processes with the highest enrichment adjusted *p*-values for each cell type, demonstrating the functional heterogeneity of granulosa and theca cells across different reproductive states (Figures 7F, G; Supplementary Tables S9, S10). The granulosa cells in anoestrous ovaries primarily exhibit the functions associated with cytoplasmic translation and viral translation. In oestrous ovaries, the granulosa cells play a key role in sterol, cholesterol, and secondary alcohol metabolism. During pregnancy, the granulosa cells are principally associated with RNA splicing. The theca cells from anoestrous, oestrous, and pregnant ovaries are primarily involved in protein translation, sterol metabolism, and the apoptotic pathway, respectively.

### 3.8 Variation in cell communication across different reproductive states

**FIGURE 8:**
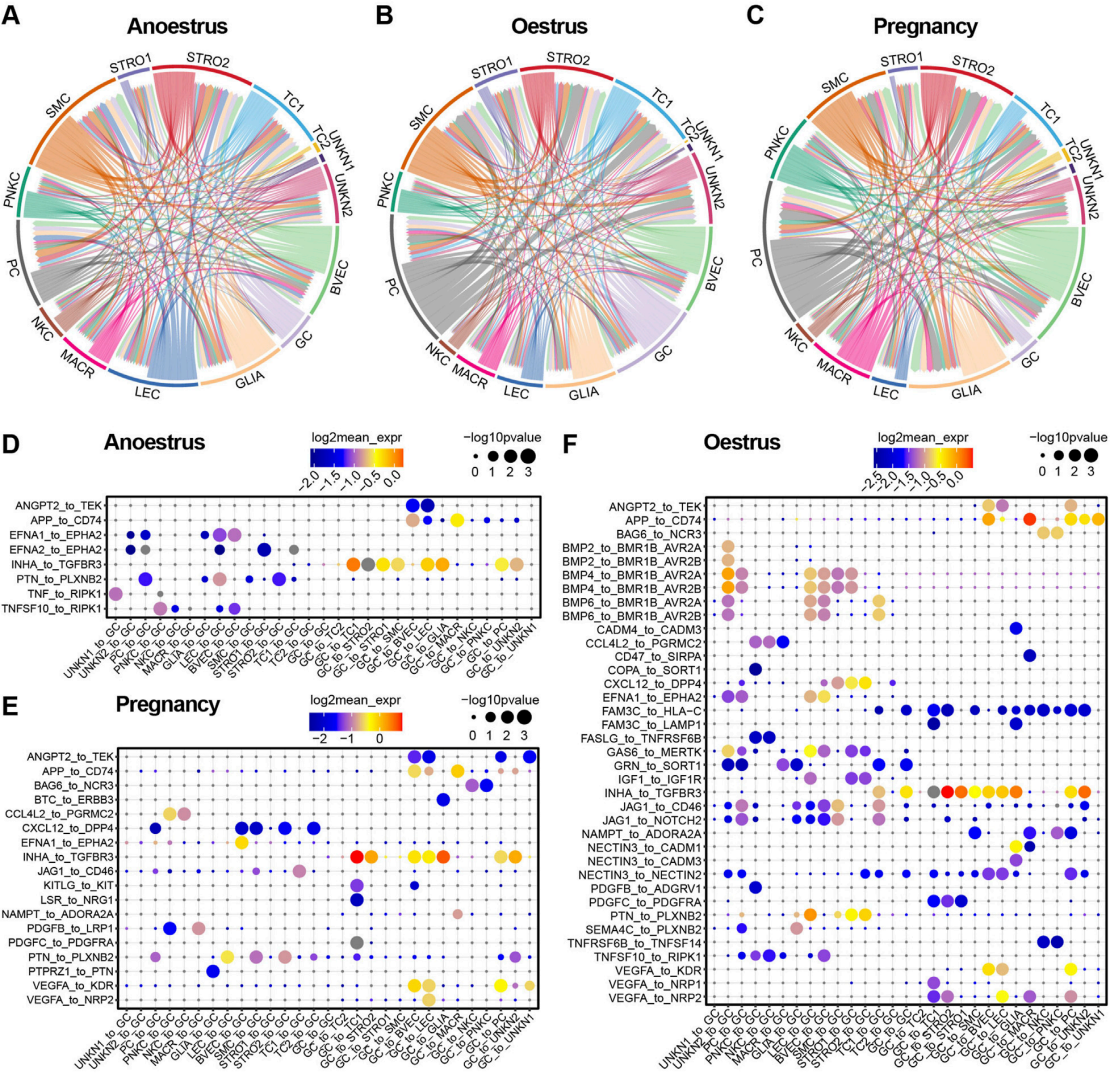
Cell-to-cell communication within yak ovaries across different reproductive states. **(A–C)** Networks manifesting the cell-to-cell communication depending on significant interactions between ligands receptors and interaction directions. Arrows originate from cell types that express ligands and point towards cell types that express corresponding receptors. STRO, stromal cell; SMC, smooth muscle cell; NKC, natural killer cell; LEC, lymphatic endothelial cell; BVEC, blood vascular endothelial cell; TC, theca cell; PC, proliferating cell; GC, granulosa cell; MACR, macrophage; PNKC, proliferating natural killer cell; GLIA, glial cell; UNKN, unknown cell. **(D–F)** Bubble plots displaying the primary ligand-receptor interactions via which granulosa cells communicate with other cell types.

To investigate the variances in cell communication across different reproductive states, the interactions between cell types were determined by assessing the expression of a receptor on one cell type and its corresponding ligand on another cell type. A cell-to-cell interaction network was established by considering the quantity of ligands and their respective receptors (Figures 8A–C; Supplementary Tables S11–S13). Unexpectedly, smooth muscle cells within yak ovaries demonstrate a close cell-to-cell correlation with other cell types during different reproductive states. The proliferative cells during oestrus and pregnancy show a greater number of intercellular junctions compared to those in the anoestrus state. Despite their small quantity, glial cells exhibit high communication with other cell types during different reproductive states. As anticipated, granulosa cells exhibit heightened communication with other cell types during oestrus compared to other reproductive states.

Bubble plots demonstrate the top significant ligand-receptor interactions through which granulosa cells communicate with other cell types across different reproductive states (Figures 8D–F). As the bubble plots indicated, there are the most ligand-receptor interactions of granulosa cells during oestrus compared to the other reproductive period, suggesting that the granulosa cells are in an active state during oestrus. Granulosa cells regulate the majority of cell types, with the exception of immune cells such as macrophages and natural killer cells, through their ligand-to-receptor interaction of INHA to TGFBR3 in all reproductive states. In contrast, the granulosa cells regulate the immune cells via their ligands APP and BAG6. During the oestrous period, granulosa cells exert control over the other cell types through their ligand FAM3C, in contrast to their role in anoestrus and pregnancy. On the contrary, the granulosa cells are principally regulated by endothelial cells, smooth muscle cells, and stromal cells, primarily through the EFNA1 and PTN ligands. The granulosa cells are regulated by other cell types through specific members of the BMP family which execute the regulatory function during oestrus.

## 4 Discussion

Various cell types have been identified within ovaries among some species, including human (Fan et al., 2019; Wagner et al., 2020; Yan et al., 2023), monkey (Wang et al., 2020b; Zhao et al., 2020a), mouse (Zhao et al., 2020b; Meinsohn et al., 2021; Morris et al., 2022), *drosophila* (Fu et al., 2020; Jevitt et al., 2020; Rust et al., 2020), and certain fish (Liu et al., 2021; Wang et al., 2021; Liu et al., 2022). Within human, monkey, and mouse ovaries, the cell types that have been identified predominantly consist of oocytes, granulosa cells, theca/stromal cells, endothelial cells, perivascular cells, smooth muscle cells, and immune cells. In the present study, similar cell types were discovered in the yak ovaries (Figure 2B). The findings suggest that there is a relative conservation of cell types in the ovaries among mammals, implying a consistent biological function within mammalian ovaries. It was difficult to distinguish theca cells from stromal cells due to a lack of marker genes with high discriminability. Despite this challenge, we successfully distinguished between theca cells and stromal cells within the yak ovary. In contrast to previous studies, our research revealed the presence of glial cells within the yak ovary, thereby expanding the diversity of cell types observed in mammalian ovaries. The majority of oocytes are typically found within the ovarian cortex, where they are enclosed within primordial follicles (Haino et al., 2018). However, differential gene expression analysis did not reveal any canonical oocyte markers from other species, hindering the identification of yak oocytes in our study.

It is important to acknowledge that ovarian cell types may exhibit species-specific signature genes. For example, Amhr2 is exclusively expressed in mouse granulosa cells, whereas BEX1 demonstrates extensive expression in human granulosa cells (Wagner et al., 2020; Wang et al., 2020a). Thecal cells emerge during the development of secondary follicles and are responsible for producing androgens and progesterone in response to LH stimulation. This process involves the upregulation of CYP11A1, CYP17A1, and STAR genes in cattle theca cells (Fukuda et al., 2009; Summers et al., 2014; Wang et al., 2019). Cells exhibiting high expression levels of STAR and CYP17A1 can be indicative of the presence of theca cells (Delgado-Rosas et al., 2009; Rojas et al., 2015). However, it is noteworthy that in other species, theca cells express high level of CYP17A1 and minimal level of CYP11A1. Consequently, a thorough analysis of the molecular characteristics of diverse ovarian cell types across different species remains crucial. In the yak ovary, the expression of CYP11A1 has been observed specifically in theca cells (Figures 3C, 4A), providing a means to distinguish them from stromal cells. This expressional distinctness has been further confirmed through immunofluorescence validation (Figure 4C). Interestingly, this high expression of CYP11A1 in theca cells of yaks aligns with that observed in cattle theca cells, suggesting the bovine specificity of theca cells compared to other species.

CYP11A1 is localized to the mitochondrial inner membrane and plays a pivotal role in catalyzing the conversion of cholesterol to pregnenolone, representing the initial and rate-limiting step in steroid hormone synthesis (Baba et al., 2018; Gu et al., 2022; Liu et al., 2023). Functionally, CYP11A1 within theca cells synthesizes androgens that serve as substrates for aromatization into estrogens by granulosa cells, which are essential for follicular growth (Xu et al., 2016). The high expression of CYP11A1 in theca cells of yak ovaries suggests a heightened demand for androgens in these cells. On the other hand, hypoxia has been shown to enhance both the expression levels of CYP11A1 and the secretion of testosterone in buffalo theca cells, a process regulated through the activation of the PI3K/AKT signaling pathway (Zhang et al., 2022). The finding implies that the elevated expression of CYP11A1 within the theca cells of yak ovaries might be induced by the hypoxic conditions prevalent on the Qinghai-Tibet plateau. This information contributes valuable insights to our understanding of ovarian function and adaptation in unique environmental contexts.

It is crucial to acknowledge the existence of heterogeneity within the same cell type. Numerous studies have demonstrated that the formation of two classes of primordial follicles occurs during early postnatal life (Frost et al., 2021). These two distinct classes arise from the breakdown of germ cell nests, which contain pregranulosa cells exhibiting varying gene expression profiles (Niu and Spradling, 2020). In complementary research, it has been observed that the inner ovarian medullary region houses germ cells surrounded by FOXL2-positive pregranulosa cells, while the outer ovarian cortical region contains migrating pregranulosa cells originating from the epithelium, initially expressing LGR5 and later transitioning to express FOXL2 (Rastetter et al., 2014). Furthermore, investigations have elucidated hierarchies among developing granulosa cell subtypes and uncovered the transcriptional programs governing their state transformation (Shi et al., 2023). In line with previous studies, our findings reveal heterogeneity within yak granulosa and theca cells, as they are classified into different subtypes (Figures 5A, 6A). These subtypes exhibit unique signature genes and biological functions, underscoring the cellular-level diversity within the ovary. The granulosa cell subtypes in the yak ovaries are primarily involved in functions such as RNA splicing, ribosome biogenesis, triphosphate biosynthetic process, translational initiation, and cell-cell adhesion (Figure 5F). On the other hand, the theca cell subtypes predominantly participate in cellular structure organization, RNA splicing, cellular respiration, triphosphate biosynthetic process, steroid metabolic process, and cytokine stimulus (Figure 6F). These findings suggest that granulosa cells and theca cells in the yak ovaries are maintained in distinct states, potentially indicating their diverse roles and functions.

The levels of steroid hormones in the serum of female vertebrates serve as indicative markers of ovarian functions across different reproductive states, exhibiting variations accordingly (Abondano et al., 2022). In our present study, we observed a higher involvement of pathways related to steroid hormone metabolism within granulosa cells and theca cells during the oestrous state compared to the other two states (Figures 7F, G), indicating increased ovarian activity during this period, which aligns with the typical oestrous manifestations. This finding is supported by a previous study that reported significantly higher mean peak estradiol-17b levels in yak during oestrus compared to anoestrus (Sarkar et al., 2006). The findings contribute to our understanding of the complex and species-specific mechanisms underlying estrogen biosynthesis and ovarian function.

Granulosa cells constitute a vital component of the follicle and play a central role in regulating oocyte development (Niu and Spradling, 2020). A recent study employing spatial transcriptomic sequencing data revealed distinct granulosa cell populations in aged versus young mice (Russ et al., 2022), underscoring the critical role of granulosa cells in maintaining female fertility and follicular cyclic homeostasis. In aged female monkeys, inactivated antioxidant pathways, increased reactive oxygen species, and apoptosis have been observed in granulosa cells, highlighting the essential connections between granulosa cell dysfunction and ovarian ageing (Wang et al., 2020b). Notably, abnormal granulosa cell function is closely associated with reproductive system disorders. For instance, mutations in the FOXL2 gene may lead to the formation of granulosa cell tumours (Weis-Banke et al., 2020; Pilsworth et al., 2021; Li et al., 2022). In patients with polycystic ovary syndrome, limited granulosa cell proliferative capacity contributes to reduced follicle maturation (Zheng et al., 2017). Extensive research in mice has demonstrated that cellular communication between oocytes and pregranulosa cells is indispensable for coordinating primordial follicle activation (Adhikari and Liu, 2009). Consequently, unraveling the molecular foundations of granulosa cell biology is of profound importance for comprehensively understanding ovarian functions and developing effective treatments for ovarian diseases.

We focused on the communication between granulosa cells and other cell types within yak ovaries (Figures 8D–F). Of particular interest, during the yak oestrus period, granulosa cells displayed more pronounced interactions with other cell types compared to the remaining reproductive states, implying the significance of cellular crosstalk and communication for successful reproductive processes. We further discovered that endothelial cells, smooth muscle cells, and stromal cells regulate granulosa cells through the interaction between BMP4 and its receptor BMPR1B. This interaction has also been observed in the ovaries of other species and is crucial for follicle survival (Kumar et al., 2020). Mutations in BMPR-1B have been linked to abnormal ovulation rates (Nilsson and Skinner, 2003). BMP4-treated ovaries exhibited a significantly higher proportion of developing primary follicles and fewer arrested primordial follicles (Nilsson and Skinner, 2003). Conversely, ovaries treated with a neutralizing antibody against BMP-4 displayed reduced size compared to controls, accompanied by a progressive loss of oocytes and primordial follicles, increased cellular apoptosis, and a decline in normal ovarian tissue morphology over time (Tan et al., 2017). In our present study, the cellular communication revealed evident regulation of granulosa cells by endothelial cells, smooth muscle cells, and stromal cells via the interaction between BMP4 and its receptor BMPR1B, suggesting that BMP4 pathway activation plays a pivotal role in promoting primordial follicle development and the transition from primordial to primary follicles in the yak follicles during oestrus.

Despite we successfully constructed a general cellular atlas of the yak ovary, it is important to acknowledge certain limitations. The inclusion of a limited number of yak ovaries as experimental samples may introduce bias if these samples do not fully represent the overall population of yak ovaries in different reproductive states. To validate our findings, future studies with larger sample sizes would be beneficial. Furthermore, it is essential to validate the reliability of the signature genes identified in our study through additional experimental methods, especially for the newly discovered marker genes. Further comprehensive investigations are needed to explore the functions of these marker genes in different cell types. Additionally, it is important to note that the cell proportions determined in our analysis may not accurately reflect the true cell proportions within yak ovarian tissue. This discrepancy could arise due to variations in sampling sites, tissue handling, and dissociation methods, which might affect different cell types differently.

## 5 Conclusion

In summary, we have successfully generated the initial cellular atlas of the yak ovaries across different reproductive states and identified the marker genes for the cell types. Furthermore, our study revealed heterogeneities within the granulosa cell and theca cell populations, as evidenced by the distinct expression patterns of genes within individual cell subtypes. Additionally, we elucidated the heterogeneities of these cell types across different reproductive states. Finally, we constructed bidirectional networks to illustrate cell-to-cell communication among different cell types across various reproductive states. These findings provide valuable insights into the mechanisms by which the ovary alters cellular phenotypes to adapt to different reproductive functions.

## Data Availability

The datasets presented in this study can be found in online repositories. The names of the repository/repositories and accession number(s) can be found in the article/Supplementary Material.
