# Supplementary figures and images for: Dynamic changes in cellular atlases and communication patterns within yak ovaries across diverse reproductive states unveiled by single-cell analysis
Source: Front Cell Dev Biol. 2024 Aug 29;12:1444706. doi: 10.3389/fcell.2024.1444706 (PMC11390571; doi:10.3389/fcell.2024.1444706)

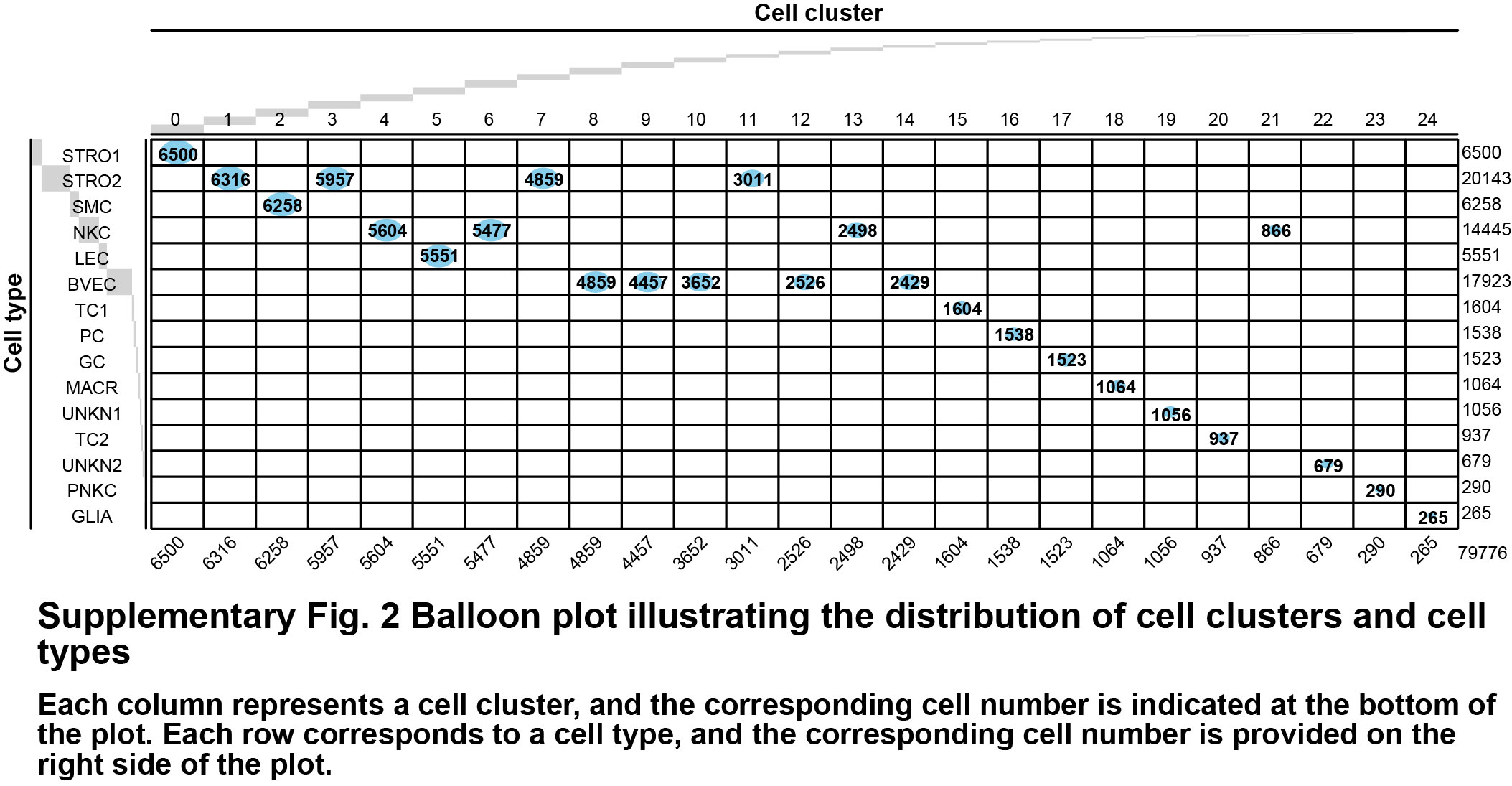

Supplement: Supplementary file 2 [file Image3.JPEG]

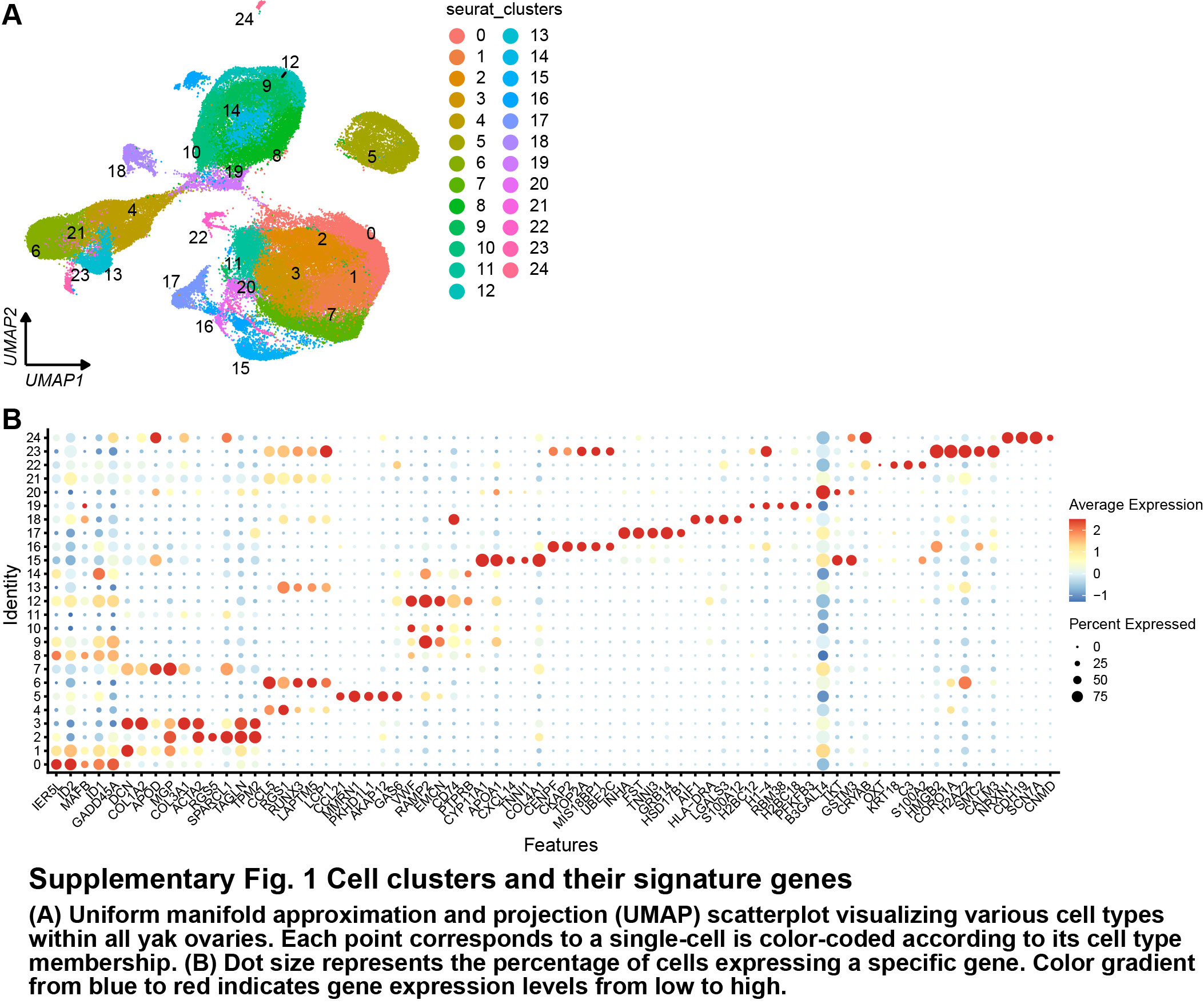

Supplement: Supplementary file 5 [file Image1.JPEG]

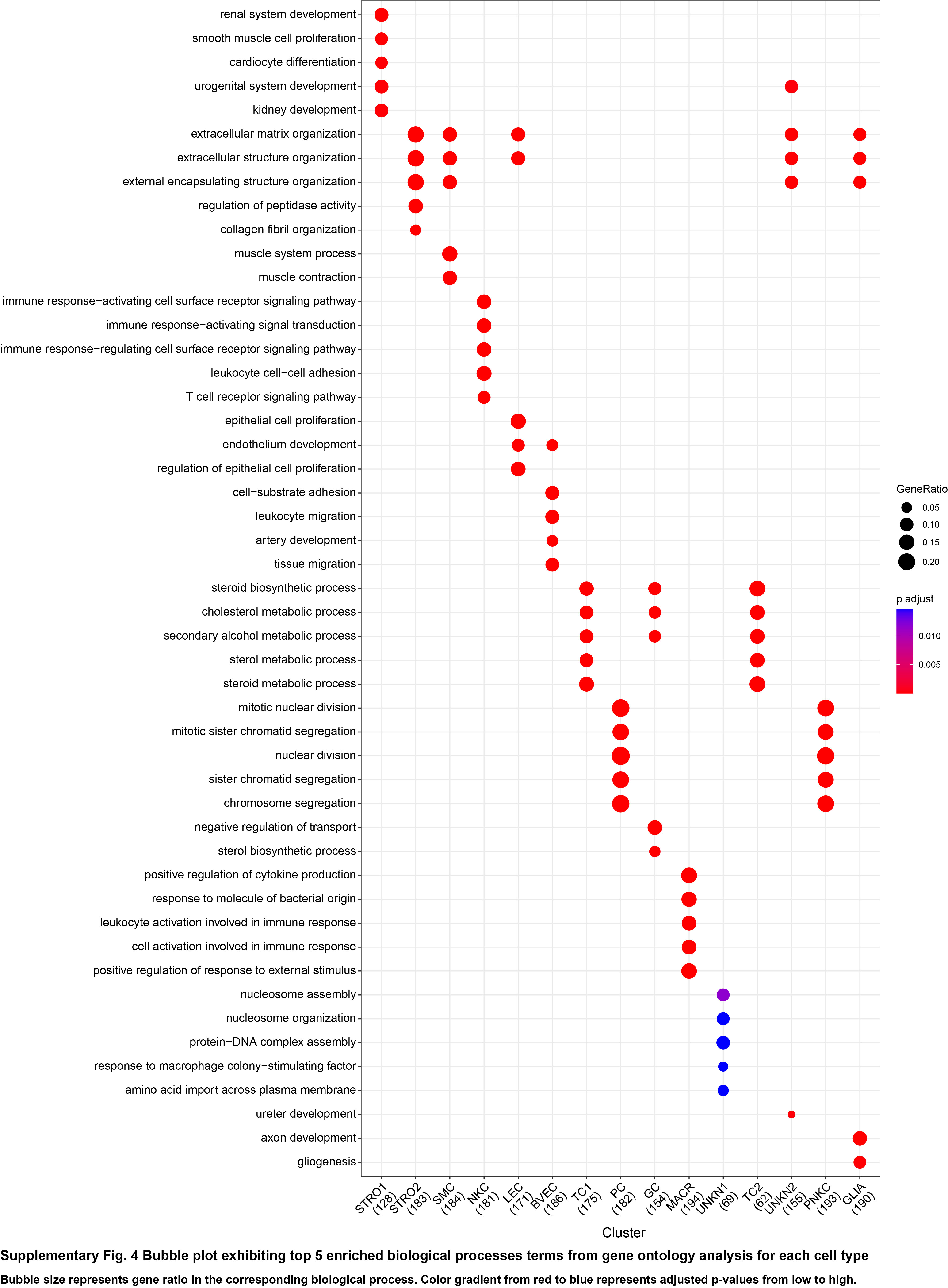

Supplement: Supplementary file 6 [file Image4.JPEG]

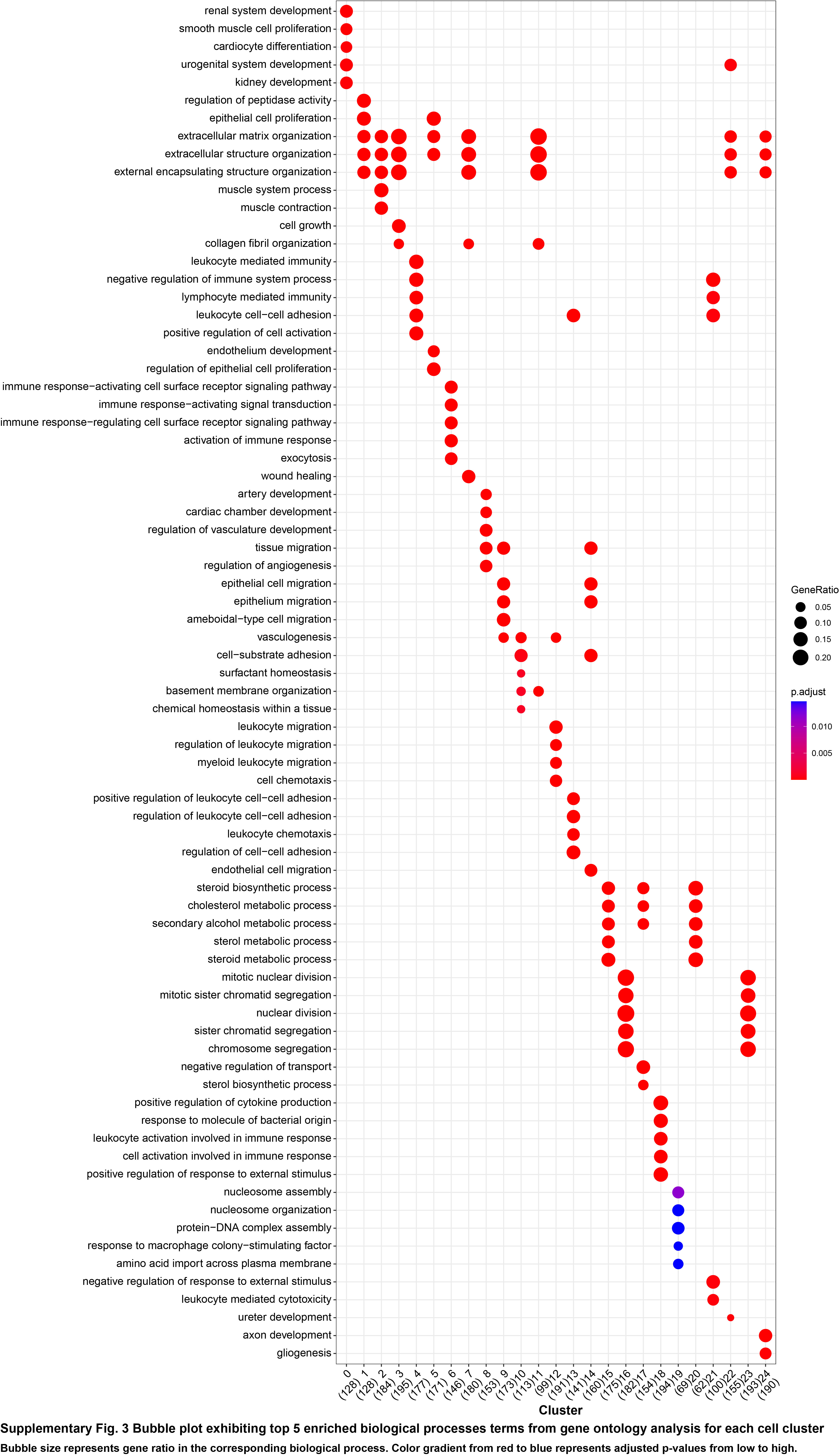

Supplement: Supplementary file 7 [file Image2.JPEG]

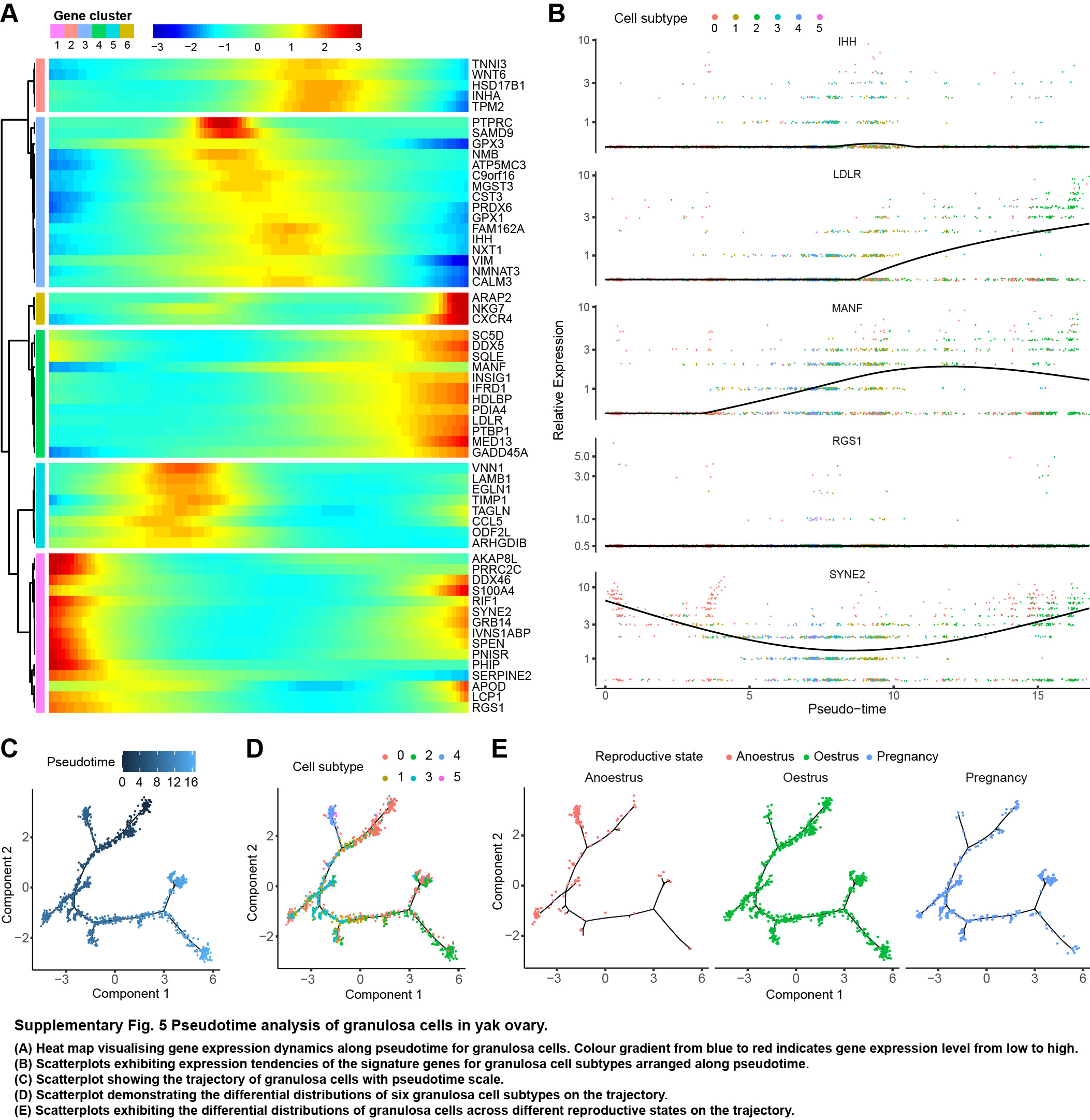

Supplement: Supplementary file 8 [file Image5.JPEG]

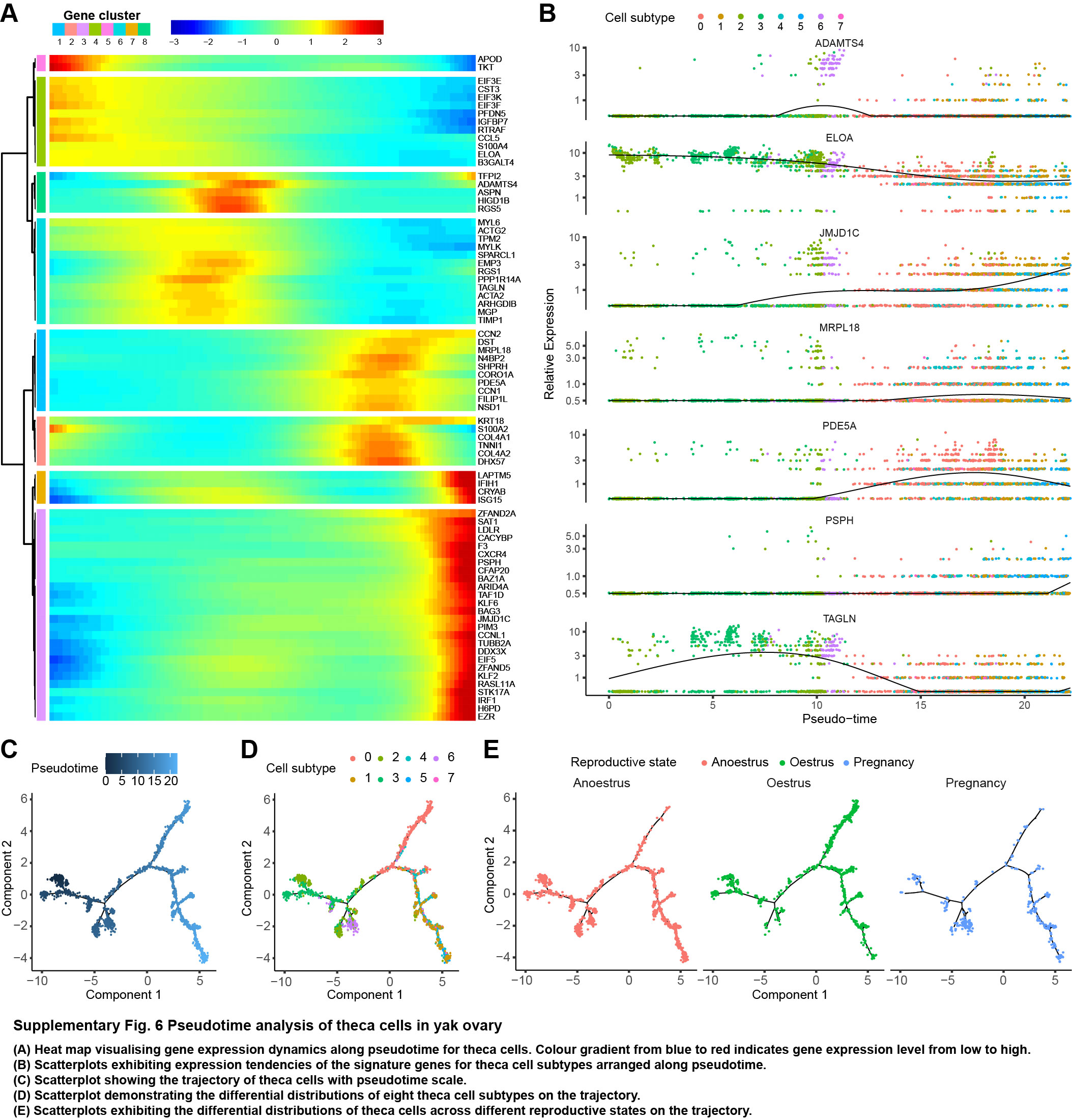

Supplement: Supplementary file 19 [file Image6.JPEG]
